# Spectral Quantum Chemistry and Infrared Resonance Library for Data-Driven Molecular Spectroscopy

**DOI:** 10.1038/s41597-026-07240-0

**Published:** 2026-04-18

**Authors:** Anirudh Krishnadas, Jatin Kansal, Nicholas E. Charron, Anita Ragyanszki

**Affiliations:** 1 Department of Distributed Algorithms and Supercomputing, Zuse Institute Berlin, Takustraße 7, Berlin, 14195 Germany; 2 Department of Mathematics and Computer Science, Freie Universitaet Berlin, Arnimallee 3, Berlin, 14195 Germany

## Abstract

Infrared (IR) spectroscopy is a fundamental tool for molecular identification and characterization, yet comprehensive IR spectral databases remain limited, particularly for small organic molecules with well-defined theoretical baselines. Here we introduce SQuIRL, the Spectral Quantum Chemistry and Infrared Resonance Library, a collection of computed IR spectra for 133,885 organic molecules. Each entry provides vibrational frequencies and intensities with near-benchmark accuracy, thereby extending the QM9 dataset by incorporating vibrational fingerprints alongside its structural and electronic descriptors. The resulting dataset enables data-driven spectrum prediction, machine-learning model training, and automated molecular identification. SQuIRL establishes a new foundation for high-fidelity, quantum-accurate infrared spectroscopy in computational chemistry. Distributed in structured HDF5 format with an accessible API, it integrates seamlessly into AI-based spectroscopy workflows and molecular discovery pipelines, offering a widely accessible resource for data-driven approaches in vibrational spectroscopy.

## Background & Summary

Infrared (IR) spectroscopy has long been central to molecular identification: from Coblentz’s first demonstration of functional-group specific bands in 1905^1^ through Lippincott’s expansion of the fingerprinting concept^2^ and the advent of Fourier-transform IR instrumentation in the mid-20th century^3^.

Due to its ease of data acquisition and its ability to provide detailed structural information, IR spectroscopy remains indispensable across domains such as pharmaceuticals^4^ and materials science^5^. Historically, IR was primarily employed to confirm the presence of select functional groups, e.g., carbonyl (*C*=*O*) stretches near 1700 cm^−1^ while the fingerprint region (400–1500 cm^−1^) remained difficult to interpret due to spectral complexity.

Extensive experimental IR spectral libraries have been compiled over decades. The NIST Chemistry WebBook^6^, for instance, includes over 16,000 IR spectra acquired under controlled conditions (gas-phase or thin film). The SDBS database^7^, maintained by Japan’s National Institute of Advanced Industrial Science and Technology, offers roughly 54,000 FT-IR spectra covering over 34,000 organic molecules. However, access is limited to image-based downloads, and data throughput is low. The Sigma-Aldrich ATR-IR library^8^ adds 19,500 proprietary spectra across diverse functional classes but is restricted by subscription access.

Despite their utility, these experimental datasets exhibit inherent limitations. Chemical coverage remains restricted to ${\mathcal{O}}(1{0}^{4}\mbox{--}1{0}^{5})$ molecules, skewed towards stable, common, and synthetically accessible compounds. Spectral gaps persist for certain functional group combinations and less-studied chemical structures. Measurement conditions, e.g., gas-phase vs. condensed-phase, introduce inconsistencies such as shifts in hydrogen-bonded or combination bands. Additionally, experimental bias and resolution variation affect peak intensity and position. Finally, limited programmatic access and licensing restrictions hinder high-throughput model training and systematic data mining, especially for proprietary datasets. To overcome these limitations, recent efforts have turned toward computational IR datasets derived from quantum chemistry or machine-learned predictors. The QMe14S^9^, an extension of QM9^10^, contains 186,000 molecules spanning 14 elements and 467 functional groups. Structures were optimized at the B3LYP/def2-TZVP level of theory, and harmonic vibrational spectra, including IR, Raman, and NMR chemical shifts, were computed. The dataset has been used to train machine learning models to predict molecular energies and spectroscopic properties directly from structure, and the increased chemical diversity was shown to improve generalization relative to QM9. Separately, Wiley’s Smart Spectra dataset^11^ includes 250,000 IR spectra generated by an AI-based engine, though accuracy remains constrained by the quality of training data, and the dataset is proprietary. Each existing dataset reflects trade-offs in accuracy (harmonic vs. anharmonic, DFT vs. higher-level theory), accessibility (open vs. licensed), and molecular scope. These limitations motivate the development of new theoretical IR resources optimized for accuracy, scale, and machine learning readiness.

There is increasing consensus within the spectroscopy and machine learning communities that large-scale, high-fidelity theoretical IR datasets are essential for data-driven molecular design. A key application is inverse structure prediction from IR spectra, long considered intractable due to spectral complexity, limited data and the general ill-posedness of solving the inverse problem. Recent work by Alberts *et al*.^12^ demonstrated that transformer models pretrained on synthetic IR spectra (>600,000 samples), conditioned on molecular formulae, can recover molecular structures directly. This capability, however, is contingent on access to large, diverse, and accurately computed spectral datasets. Beyond structure prediction, such datasets enable inverse molecular design, which includes generating candidate molecules that satisfy a specified IR profile. Achieving this requires not only spectral accuracy but also consistency in format, resolution, and integration with programmatic workflows.

In this work, we present **SQuIRL** (Spectral Quantum Chemistry and Infrared Resonance Library)^13^, a high-accuracy infrared spectral dataset comprising ≈134,000 small organic molecules from the QM9 database. Starting from QM9’s pre-optimized geometries (at the B3LYP/6-31G(2df,p) level), we computed harmonic vibrational frequencies and IR intensities using the *ω*B97X-D functional with the aug-cc-pVTZ basis set. This range-separated hybrid functional and diffuse triple-zeta basis provides improved accuracy for vibrational properties, particularly in capturing dispersion and low-frequency modes. The dataset is publicly released in convenient Hierarchical Data Format Version 5 (HDF5) format with a documented API, supporting high-throughput integration into machine learning workflows. SQuIRL provides computed IR spectra generated using a consistent computational protocol and made available in standardized HDF5 format. The following sections describe the computational protocol, benchmarking, diversity analysis, and data access.

Functional group analysis confirms that the dataset spans a wide chemical space, covering common groups such as alcohols, ethers, and amines, as well as less frequent but chemically important classes such as nitro compounds, ureas, and guanidines. Distributions of dipole moments, molecular sizes, and zero-point vibrational energies demonstrate a comprehensive sampling of electronic and vibrational features without bias toward any single chemical class. Similarity analysis further indicates structural diversity even within individual functional groups. The release includes molecules spanning diverse functional groups, with accompanying structural, thermochemical and spectroscopic data and metadata.

Another key distinction is that SQuIRL provides raw, unprocessed harmonic frequencies and intensities. This enables researchers to apply their own post-processing (e.g., Lorentzian or Gaussian broadening functions, scaling factors, or anharmonic corrections) depending on workflow requirements. We note that this harmonic treatment follows common practice in high-throughput spectroscopy workflows, and that more accurate, but substantially more expensive, anharmonic methods can be applied to selected systems when closer agreement with experiment is required. In addition, SQuIRL includes associated molecular descriptors such as thermochemistry, polarizabilities, dipole moments, and SMILES strings, broadening its usability beyond spectroscopy. Finally, SQuIRL is designed as a growing, evolving resource; additional molecules and spectral data will be incorporated in future updates, further expanding its chemical diversity and methodological consistency.

The dataset is openly available for download^13^ by researchers, academic groups, and industry practitioners. The dataset is available for integration into computational workflows and may be applied in data-driven analyses.

## Methods

All quantum chemical calculations were performed using the Gaussian 16 software package^14^ at the *ω*B97X-D/aug-cc-pVTZ level of theory. The *ω*B97X-D functional is a range-separated hybrid density functional that incorporates empirical dispersion corrections (denoted by “-D”), specifically the *D*2 version of Grimme’s dispersion model^15^. This correction enhances the treatment of long-range van der Waals interactions, improving the accuracy for noncovalent systems, conformational energetics, and vibrational properties. The basis set used, aug-cc-pVTZ, is part of the correlation-consistent family developed by Dunning^16^. The cc stands for correlation-consistent, indicating that the basis set was systematically constructed for accurate post-Hartree-Fock correlation energy convergence. “VTZ” refers to the triple-zeta quality in the polarization-augmented valence space, which balances accuracy and computational cost. The “aug” prefix denotes the inclusion of diffuse functions on all atoms, which are essential for properly describing electron density in systems with lone pairs, anions, or delocalized charge distributions, which are critical for reliable prediction of vibrational frequencies and infrared intensities. All calculations were carried out in the gas phase at standard conditions (298.15 K and 1 atm). Harmonic vibrational frequency analyses were used to confirm the nature of stationary points (characterized by one or more modes with non-trivial imaginary frequencies) and to extract zero-point energies and thermochemical corrections. For each molecule, a discrete harmonic IR spectrum consisting of peak positions (wavenumbers) and associated intensities was generated. Only 0.075% (101 molecules) of the 133,885 molecules showed non-trivial imaginary harmonic frequencies. These cases are inherited from the QM9 starting geometries and indicate structures that are not local minima at the present level of theory; the imaginary modes most commonly reflect transition-state-like geometries, rather than numerical artifacts. To maintain compatibility with QM9 indexing and ensure transparent reporting, these entries are retained and are explicitly flagged via a “warning” field in the dataset metadata. Users may therefore filter out molecules flagged by “warning” if their application requires strictly bound minima; given their low prevalence, these cases do not affect the dataset statistics or validation results. In addition, the frequency analyses provide vibrational symmetries and force constants, and the dataset also includes dipole moments and polarizability tensors.

## Data Records

**Fig. 1:**
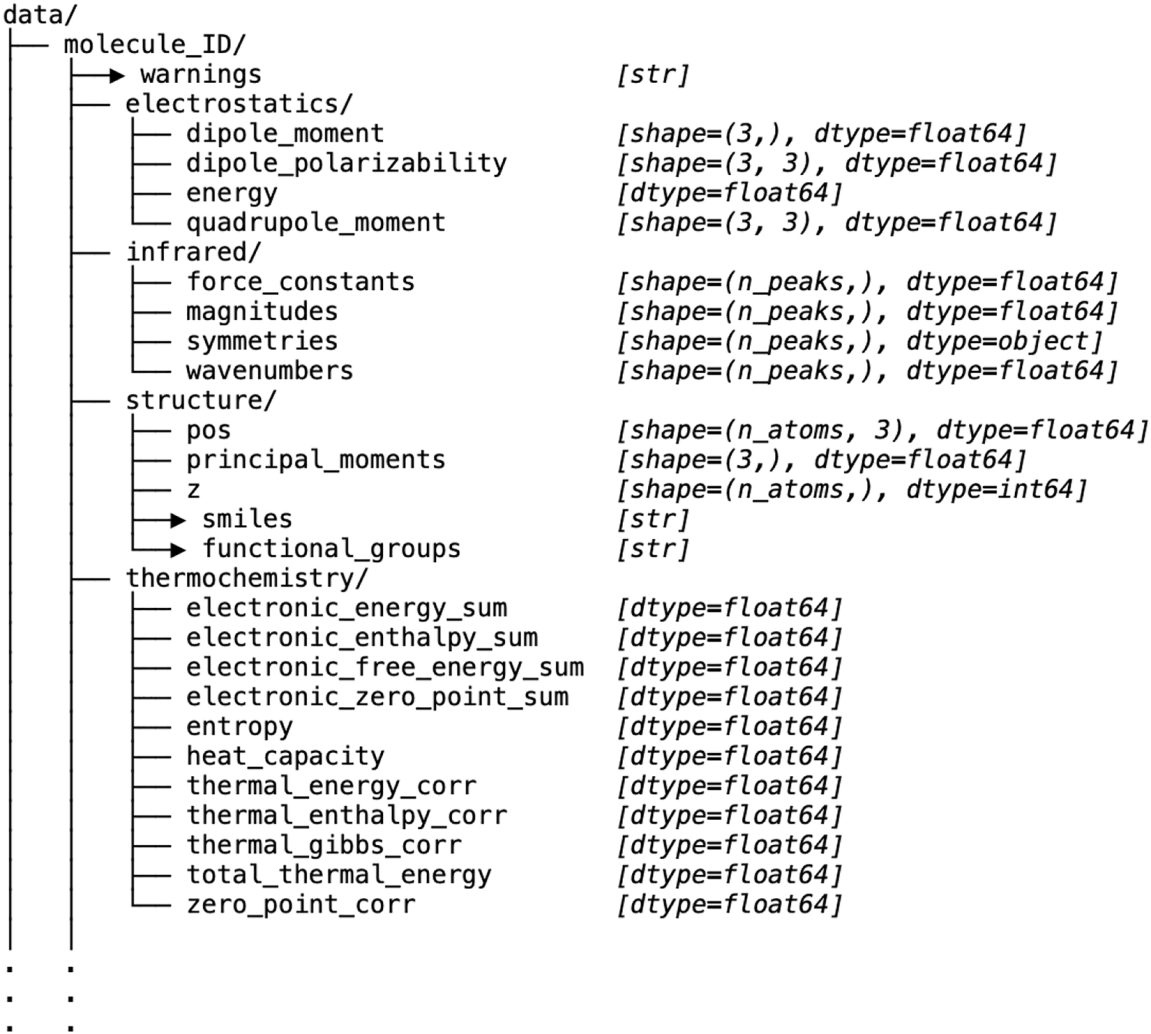
HDF5 record structure. Arrows indicate metadata/H5 “attribute” records, while all other entries are numerical records.

The hierarchical data format (HDF5) structure is illustrated in Fig. 1. Molecules are indexed analogously to the QM9 database, with infrared spectra, structural, and energetic information provided alongside SMILES representations. Additional quantum chemical descriptors, including dipole polarizabilities, quadrupole moments, dipole moments, and total energies, are also included. This organization facilitates seamless integration with widely used computational frameworks such as PyTorch, TensorFlow, RDKit, and ASE, supporting both conventional workflows and modern machine learning applications.

The **SQuIRL** dataset is publicly hosted on Figshare (10.6084/m9.figshare.30734843)^13^. It includes all molecular structures together with their computed harmonic IR spectra and the following physicochemical properties: electrostatics, dipole moments, dipole polarizabilities, raw vibrational wavenumbers and intensities, and electronic zero-point vibrational energies. All data are provided in a ready-to-use format and are openly accessible for research and development purposes.

In addition to the raw dataset, we provide a comprehensive README file that outlines all necessary instructions for accessing and utilizing the data. This document includes step-by-step guidelines for reading files in HDF5 format, example scripts for integration with widely used Python libraries such as RDKit, ASE, and TensorFlow/PyTorch, as well as a detailed description of all metadata attributes. The HDF5 file structure stores molecular geometries, SMILES representations, harmonic vibrational spectra, and key electronic properties, including dipole moments and zero-point vibrational energies. A summary of the molecular properties available in the dataset, along with their respective units, is provided in Table 1. The warning field records quality check outcomes such as detection of imaginary frequencies or parsing anomalies, enabling straightforward filtering in downstream workflows. Furthermore, per-species atomization energies are included to support benchmarking, model training, and downstream computational analysis.

**Table 1:** Summary of molecular properties provided in the SQuIRL dataset.

Property	Units
SMILES strings	—
Molecular geometries (Cartesian)	Å
Functional groups	—
Principal moments of inertia	amu Å^2^
Force constants (normal modes)	mDyne/Å (= 0.1 N/m)
Harmonic IR intensities	km mol^−1^
Harmonic vibrational wavenumbers	cm^−1^
Point group symmetry	—
Dipole moment	Debye
Dipole polarizability	a.u. (bohr^3^)
Quadrupole moment	Debye ⋅ Å
Electronic energy (SCF)	Hartree
Zero-point vibrational energy (ZPVE)	Hartree
Thermal correction to energy	Hartree
Thermal correction to enthalpy	Hartree
Thermal correction to Gibbs free energy	Hartree
Sum of electronic and ZPVE	Hartree
Sum of electronic and thermal energies	Hartree
Sum of electronic and thermal enthalpies	Hartree
Sum of electronic and thermal free energies	Hartree
Heat capacity *C*_*V*_ (thermal analysis)	cal mol^−1^ K^−1^
Warnings / notes	—
Atomic numbers (*Z*)	—

**Notes:** (i) 1 a.u. of polarizability $\,=\,{a}_{0}^{3}\,=$ bohr^3^ ≈ 0.14818471 Å^3^. (ii) Gaussian reports quadrupoles in Debye ⋅ Å (sometimes called “Buckingham-like”); if you prefer Buckinghams, 1 B = 10^−26^ esu cm^2^. (iii) Gaussian’s thermochemistry section also prints *E*_thermal_ and *C*_*V*_ in kcal/mol and cal mol^−1^ K^−1^ respectively; we store energies in Hartree for consistency. (iv) All these properties are calculated at 298.15 K with 1 atm pressure. (v) Individual Atomization energies of ’C’,’O’,’H’,’N’ and ’F’ are provided in Hartree.

The dataset distribution also includes a companion metadata CSV file that summarizes key per-molecule attributes, including the HDF5 path, SMILES representation, functional groups, warnings, number of atoms, number of vibrational peaks, dipole components and magnitude, principal moments of inertia, and selected electronic and thermochemical quantities. This tabular index enables rapid filtering, querying, and exploratory analysis without requiring traversal of the full HDF5 hierarchy.

## Technical Validation

The dataset comprises a broad range of vibrational modes, including C-H, O-H, N-H, C=O, C-N, as well as mixed-mode couplings, with computed frequency shifts consistent with benchmark experimental measurements. It comprises approximately 134,000 structurally diverse small molecules, up to nine heavy atoms, thereby ensuring wide coverage of chemical space, including numerous functional groups and heteroatom-containing species.

Table 2 presents a comparison of SQuIRL with other recent computational and experimental IR databases. Experimental databases, such as NIST and SDBS, remain invaluable for direct spectral validation; however, they are limited in size and accessibility for large-scale, machine learning driven workflows. By contrast, computational datasets enable high-throughput and uniform coverage. Among them, the recently reported QMe14s collection is of particular relevance. While QMe14s is larger in molecular count (186,000 molecules) and includes multiple spectroscopic properties (IR, Raman, NMR), its underlying calculations employ the B3LYP/def2-TZVP level of theory.

**Table 2:** Comparison of infrared (IR) spectroscopy databases with the present SQuIRL dataset. Counts and details are taken from the cited sources. Here, **C** denotes computationally calculated data and **E** denotes experimentally measured data.

Database	Molecules	Properties included	Data source	Comp. level	Format	Access
**SQuIRL**	133,885	IR, Thermochemistry, Polarizability, Dipole, SMILES	C	*ω*B97X-D (aug-cc-pVTZ)	HDF5	Free
QMe14s^9^	186,102	IR, Raman, NMR, Thermochemistry	C	B3LYP (def2-TZVP)	HDF5	Free
NIST Chemistry WebBook^6^	~16,000	IR, UV/Vis, MS; Thermochemistry	E	N/A	Web (JCAMP for some)	Free
SDBS (NIMS)^7^	~34,000	IR, NMR, MS, ESR, Raman	E	N/A	Web	Free (some restrictions)
Sigma-Aldrich ATR-IR^8^	~19, 503	IR	E	N/A	Proprietary PDF	Licensed

In comparison, **SQuIRL** employs the range-separated hybrid functional *ω*B97X-D in combination with the aug-cc-pVTZ basis, which provides a more balanced description of both short- and long-range interactions. This choice has direct implications for vibrational accuracy and transferability. Benchmarking studies of vibrational scaling factors report that B3LYP/def2-TZVP harmonic frequencies can typically be corrected with a scale factor of 1.0044, resulting in residual root-mean-square deviations (RMSDs) of ~26–27 cm^−1^ ^17^ whereas *ω*B97X-D/aug-cc-pVTZ employs a scale factor near 0.9917 and yields RMSDs of ~30–33 cm^−1^ after scaling^18,19^. Despite this difference, broader functional benchmarks indicate that modern range-separated hybrids such as *ω*B97X-D provide a more balanced accuracy across diverse molecular properties (thermochemistry, noncovalent interactions, charge transfer), even though their scaled harmonic vibrational RMS errors are comparable to or slightly higher than those of B3LYP^20^. The merit of SQuIRL therefore lies in providing IR intensities, dipole moments, and vibrational properties at a consistently higher level of electronic-structure theory across all molecules, which is particularly valuable for hydrogen-bonded, charge-transfer, and electronically sensitive systems where B3LYP exhibits known systematic errors. This spectroscopic fidelity, combined with comprehensive metadata and standardized data formatting, positions SQuIRL as complementary to QMe14s: the latter remains well-suited for large-scale screening and rapid prototyping, whereas SQuIRL serves machine-learning applications where physical accuracy of IR observables is critical. Previous studies have shown that datasets computed at higher and more uniform levels of electronic-structure theory improve model robustness and transferability; SQuIRL is constructed explicitly to support such downstream spectroscopy-focused machine-learning workflows.

**Fig. 2:**
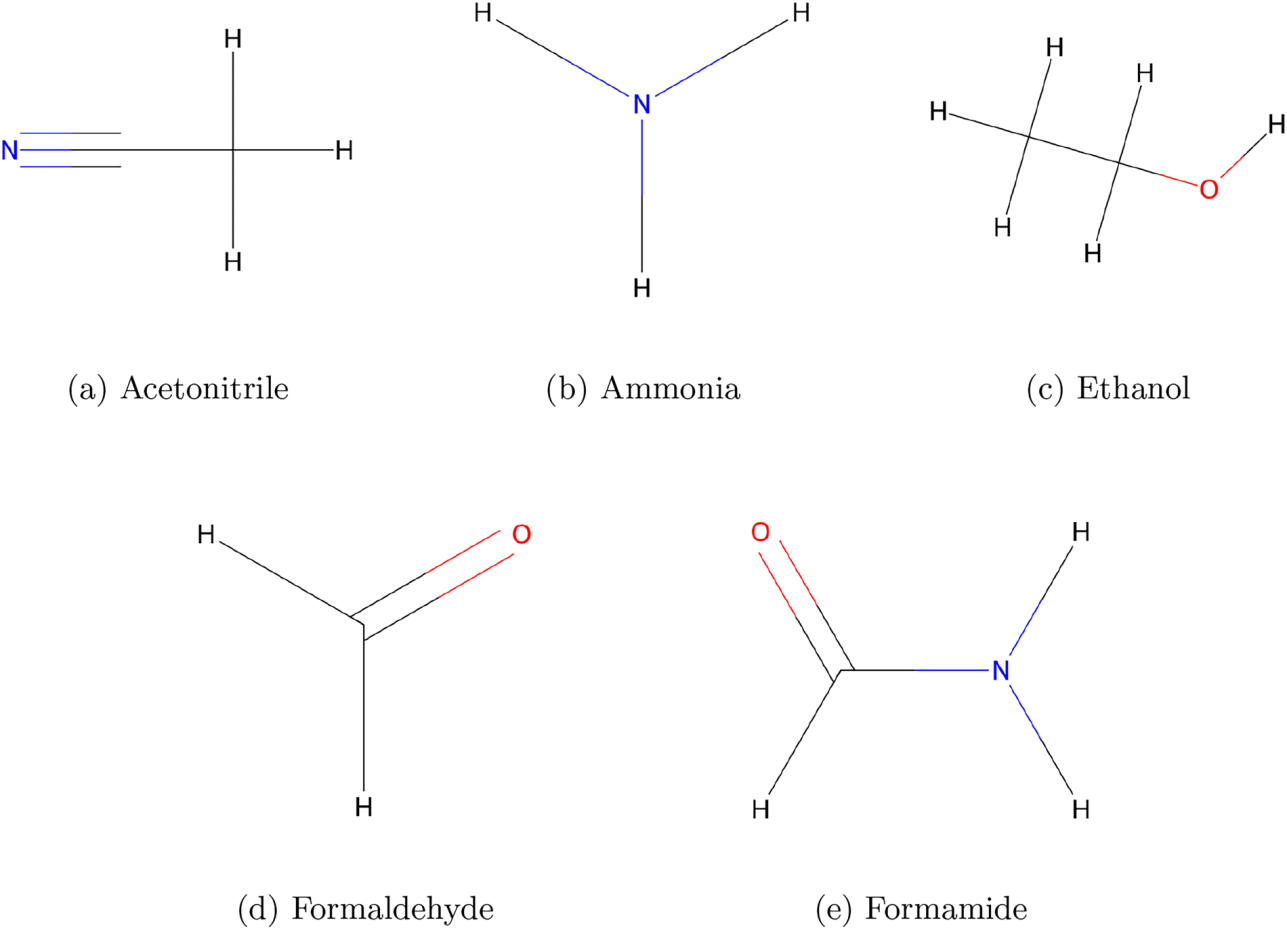
Molecular structures optimized for benchmarking.

The database molecules were pre-optimized at the B3LYP/6-31G(2df,p) level (QM9). In this work, we computed harmonic vibrational frequencies at a higher level of theory to benchmark against Coupled Cluster Singles and Doubles (CCSD)^21^ with the aug-cc-pVTZ basis set (Optimization and frequency), as well as against available experimental IR spectra. The objective of this benchmark was to assess the accuracy of the present dataset (SQuIRL) in reproducing vibrational modes across a range of functional groups. To this end, we selected five representative molecules: acetonitrile, ethanol, ammonia, formamide, and formaldehyde covering characteristic functional groups such as C-N, N-H, O-H, and C=O modes (see Fig. 2).

**Fig. 3:**
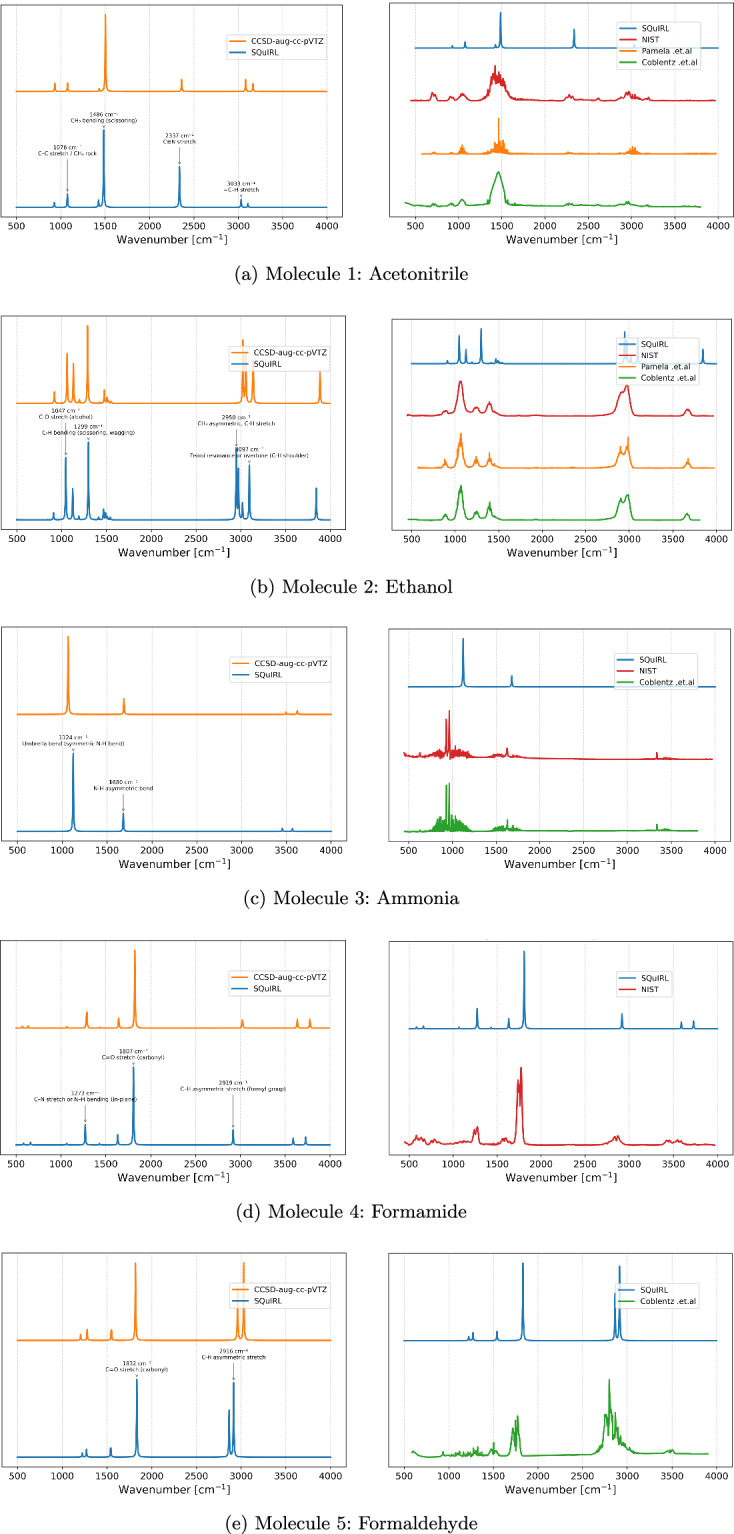
Computed and experimental IR spectra for five small organic molecules.

Figure 3 shows the benchmark results for these five representative cases. In each panel, the left plots compare SQuIRL (blue) with CCSD/aug-cc-pVTZ (orange), while the right plots compare SQuIRL with experimental spectra extracted from the NIST database^22^ (Coblentz *et al*.^23^) and additional literature sources (e.g., Pamela *et al*.^24^). Major vibrational assignments are annotated in the left panels. The experimental, CCSD-calculated, and SQuIRL spectra shown in the panel have been scaled using min-max normalization to enable a clearer visual comparison of the peak positions.

In all comparisons, we applied a uniform full width at half maximum (FWHM) of 4 cm^−1^, consistent with the GaussView^25^ default in our setup. While tuning the broadening could improve agreement with specific experimental datasets, we retained this setting for consistency across all molecules.

It is important to emphasize the computational scaling considerations underlying the construction of SQuIRL. The present hybrid DFT calculations scale formally as ${\mathcal{O}}({n}^{3})$ with system size, whereas canonical CCSD scales as ${\mathcal{O}}({n}^{6})$. Even for the small systems tested here, a single CCSD harmonic frequency calculation required on the order of 2–3 core-years per molecule, rendering a systematic CCSD benchmark across the full dataset computationally prohibitive. At the DFT level, inclusion of explicit anharmonic corrections further increases the computational cost substantially. For example, in our benchmark for NH_3_ at the *ω*B97X-D/aug-cc-pVTZ level, an anharmonic frequency calculation required approximately 55 CPU minutes compared to 1.5 CPU minutes for the corresponding harmonic job (a ~37-fold increase), while reducing the mean absolute error relative to experiment^26^ from ≈5% to ≈1%. While this improvement is significant for small systems, extending such calculations systematically to thousands of molecules would be computationally impractical.

Nevertheless, harmonic DFT frequencies reproduce CCSD harmonic results with typical deviations of 10–15 cm^−1^, consistent with prior benchmarks, and experimental IR fundamentals within the customary 20–40 cm^−1^ accuracy associated with hybrid DFT methods^27^. Taken together, these results support the use of the present DFT harmonic framework as a balanced and computationally tractable choice for constructing large-scale infrared spectroscopy datasets.

To further characterize the diversity and chemical coverage of the dataset, we performed a systematic functional and bonding pattern analysis. Beyond structural enumeration, this analysis highlights the spread of chemical structures and their associated electronic and vibrational properties, which are of particular importance for developing machine learning models that rely on balanced and representative training data. In addition, the ability to query the dataset by functional or bonding groups provides a flexible route for constructing chemically meaningful training/test splits.

**Fig. 4:**
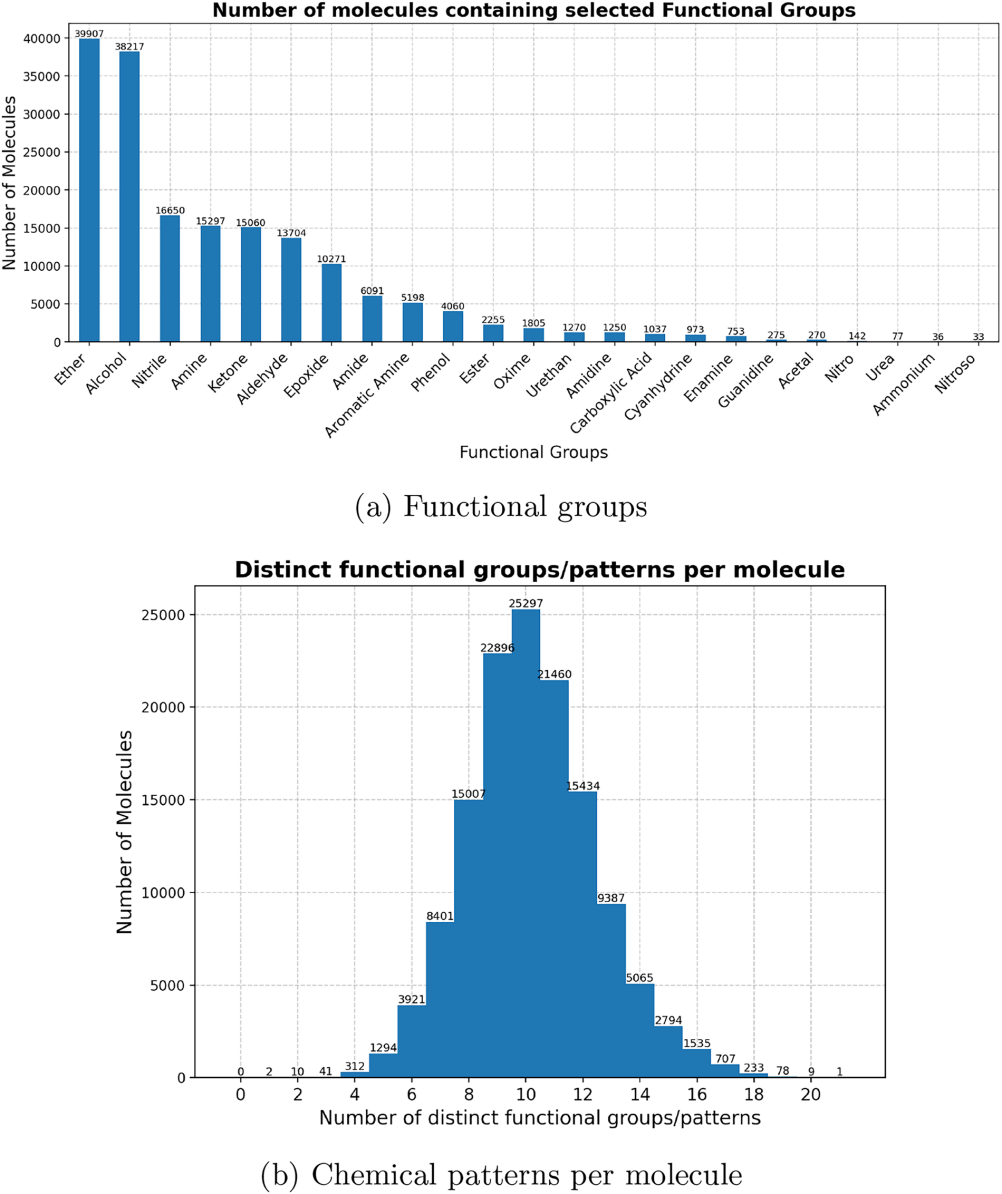
Diversity of functional groups and substructure patterns across the dataset. Panel **(a)** shows the distribution of common functional groups, while panel **(b)** gives the number of distinct patterns identified per molecule.

**Fig. 5:**
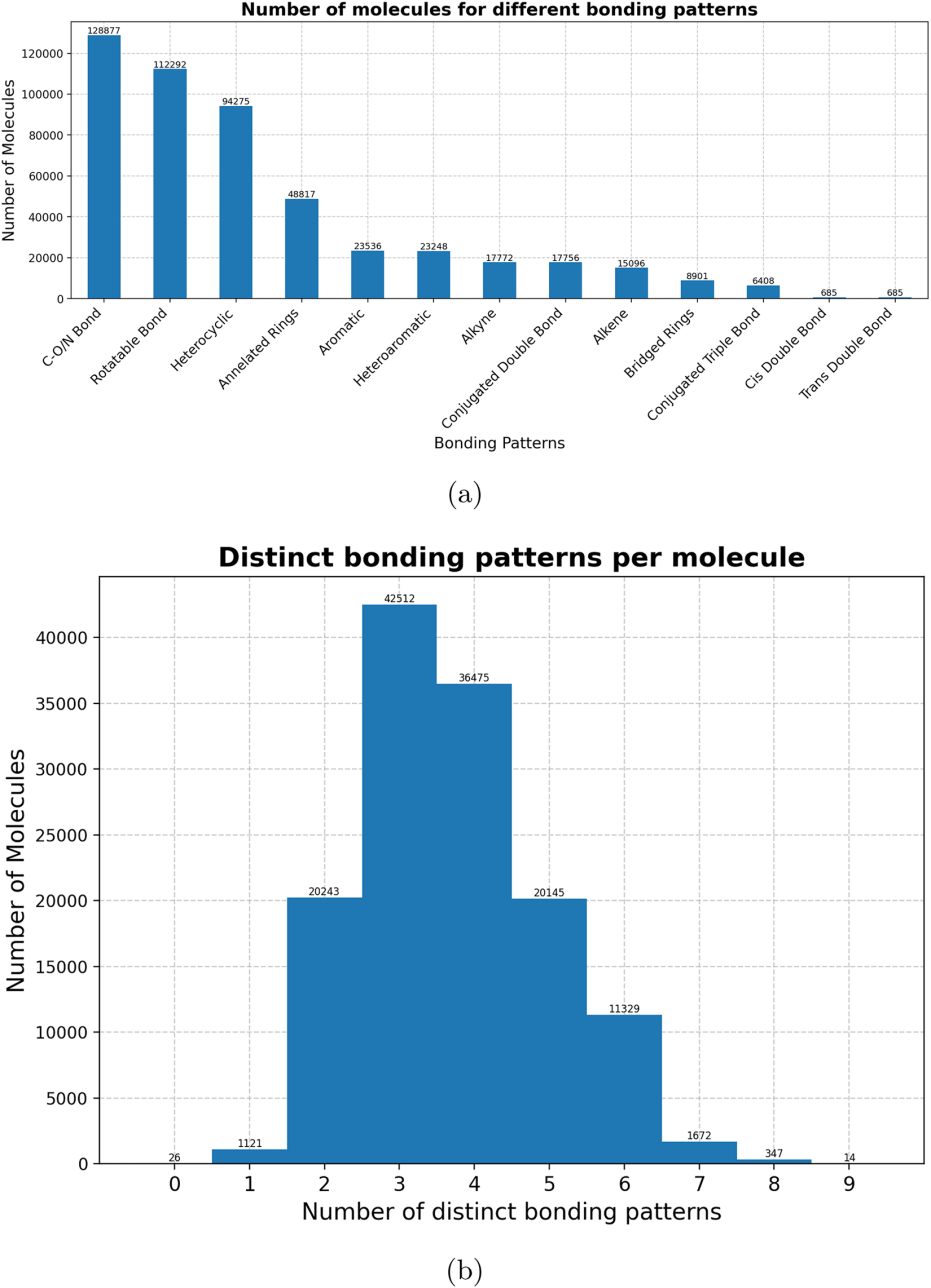
Bonding pattern diversity across the dataset. Panel **(a)** shows the prevalence of common bonding structures, while panel **(b)** illustrates the distribution of distinct bonding structures per molecule.

We employed the SMARTS library^28^, which provides curated pattern definitions for functional groups, substructures, and common chemical structures. Substructure matching was carried out with RDKit, yielding the location and frequency of matches for each pattern in every molecule. Figs. 4 and 5 summarize the distribution of functional groups and bonding motifs across the dataset.

From Fig. 4(a), ethers and alcohols emerge as the most prevalent groups (nearly 40,000 molecules each), followed by nitriles, amines, ketones, and aldehydes. Nonetheless, the dataset retains significant coverage of less common functional groups, including ureas, guanidines, nitro compounds, and nitroso species, ensuring that rare but chemically relevant functionalities are also represented. The histogram in Fig. 4(b) shows that most molecules contain between 8 and 12 distinct functional patterns, with the distribution centred at 10. This breadth demonstrates that the dataset spans multi-functionalized molecules, which is crucial for realistic benchmarking of vibrational couplings and IR activity.

Bonding pattern statistics (Fig. 5) reveal that *C*−*O* and *C*−*N* bonds are the most common, alongside rotatable bonds and heterocycles. Importantly, while aromatic and conjugated double bonds occur frequently, the dataset also includes more specialized structures such as bridged rings and cis/trans-double bonds, which are critical for testing algorithms sensitive to electronic delocalization and conformational flexibility. The majority of molecules contain 2 − − 4 distinct bonding patterns, highlighting the dataset’s balance between structural simplicity and chemical complexity.

Beyond connectivity, we analyzed molecular properties linked to electrostatics and nuclear motion, including dipole moments, molecular size, and zero-point vibrational energy (ZPVE). Imbalances in the distribution of these quantities can alter IR intensities and, more broadly, introduce systematic biases that impact the performance and generalization of downstream machine learning models.

**Fig. 6:**
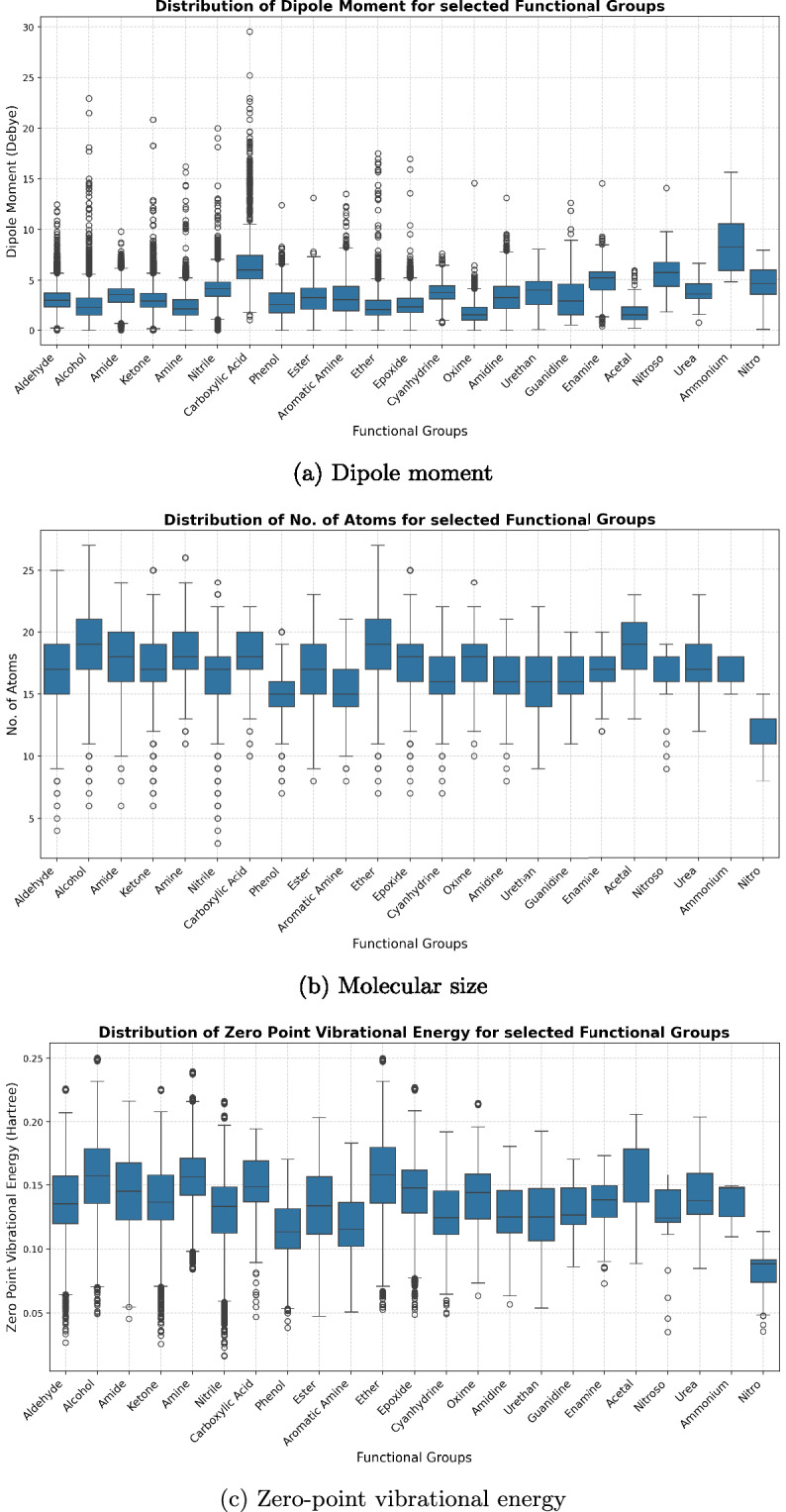
Distributions of key electrostatic and vibrational properties across molecules containing selected functional groups. Together, dipole moments, molecular sizes, and ZPVEs illustrate the dataset’s coverage of diverse chemical and spectroscopic regimes.

Figure 6(a) presents dipole moment distributions for molecules containing selected functional groups. As expected, strongly polar groups such as nitriles, nitro, and ammonium species display broad distributions with higher median values (5−7 D), while ethers and phenyls cluster near lower dipole ranges (<3 D). This confirms that the dataset samples both highly polar and weakly polar systems, preventing skew in electrostatic response.

Molecular size distributions (Fig. 6(b)) show that most functional groups span 10 − 20 atoms, with slightly larger averages for aromatic amines and guanidines due to their extended ring or chain systems. This indicates that functional diversity is not confined to the smallest molecules but extends into moderately complex systems as well.

Zero-point vibrational energies (Fig. 6(c)) reveal consistent group-wise trends: functional groups with heavier atoms exhibit lower ZPVE ranges, while groups dominated by X-H stretching modes (alcohols, amines, amides) display higher ZPVE distributions. The median ZPVE values cluster around 0.12-0.18 Hartree, with tails extending to 0.25 Hartree for highly flexible species. These features confirm that vibrational contributions are broadly and evenly sampled across chemical classes, supporting generalization of ML models to thermodynamic and spectroscopic observables.

**Fig. 7:**
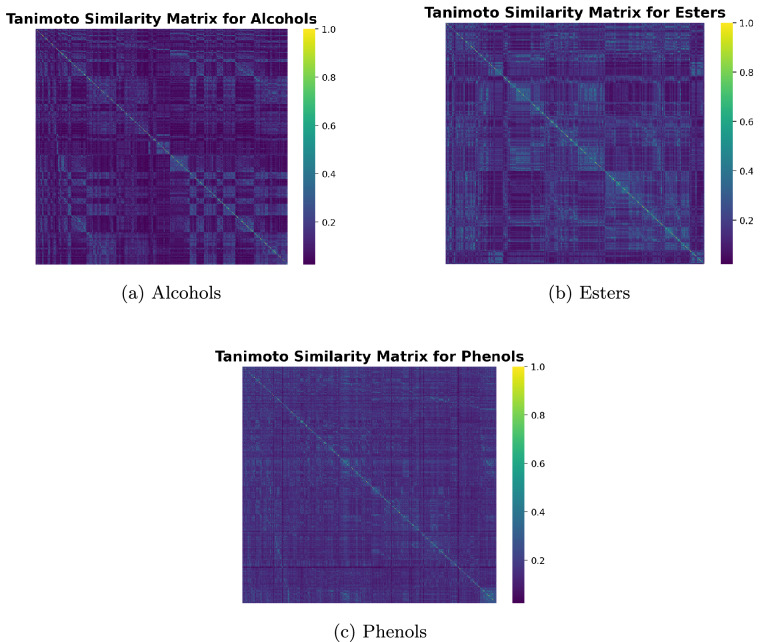
Tanimoto similarity matrices for selected functional groups.

Finally, we examined chemical redundancy within functional subgroups using Tanimoto similarity matrices derived from Morgan fingerprints. Specifically, we employed the GetMorganFingerprintAsBitVect method (radius = 2, *n*_bits_ = 1024) from the rdkit.Chem.rdMolDescriptors module to generate binary fingerprints, and computed pairwise similarities with the TanimotoSimilarity method from rdkit.DataStructs, as implemented in RDKit^29^. Representative cases for alcohols, esters, and phenols are shown in Fig. 7. Although small blocks of high similarity are visible (e.g., short alcohol chains, closely related ester derivatives), the majority of pairwise similarities are significantly below unity, demonstrating that the dataset includes chemically distinct variants even within a single functional category. This structural diversity prevents over-representation of highly correlated molecules and ensures that machine learning models trained on these subsets are not trivially biased toward repetitive chemical motifs.

## Data Availability

The SQuIRL dataset is publicly available on Figshare at 10.6084/m9.figshare.30734843. Together with the dataset, we provide a self-contained Jupyter notebook that shows how the dataset can be loaded, and the records can be accessed and inspected.  A companion CSV file is also available to enable filtering.

## Code availability

Together with the dataset, we provide a self-contained Jupyter notebook that shows how the dataset can be loaded and the records can be accessed and inspected.

## References

[1] Coblentz, W. W. Investigations of Infra-Red Spectra, Carnegie Institution of Washington Publication. vol 35, 65 & 97 (Carnegie Institution of Washington, Washington, D.C., 1905).

[2] Lippincott, E. R. The limitations and advantages of infrared spectroscopy in patent problems. *Journal of the Patent Office Society* **45**, 380–415 (1963).

[3] Griffiths, P. R. & de Haseth, J. A. Fourier Transform Infrared Spectrometry. 2nd edn. (Wiley-Blackwell, Hoboken, NJ, 2007).

[4] Kiefer, J. & Cheng, R. Analysis of tablets using attenuated total reflection infrared spectroscopy: Spectral artifacts and their correction. *American Pharmaceutical Review* **27**(5), 30–33 (2024).

[5] Wetzel, W. How FT-IR spectroscopy advances nanomaterial research. *Spectroscopy Online* (2025).

[6] National Institute of Standards and Technology. Nist chemistry webbook. https://webbook.nist.gov/. Accessed: 2025-07-22 (2025).

[7] National Institute of Advanced Industrial Science and Technology (AIST). Sdbs – spectral database for organic compounds. https://sdbs.db.aist.go.jp/sdbs/ (1997).

[8] Sigma-Aldrich Co. LLC and Wiley Science Solutions. Sigma-aldrich library of atr-ir spectra. DVD spectral library (2019).

[9] Yuan, M., Zou, Z., Luo, Y., Jiang, J. & Hu, W. Qme14s: A comprehensive and efficient spectral data set for small molecules. *The Journal of Physical Chemistry Letters* **16**(16), 3972–3979 (2025). 40223330 10.1021/acs.jpclett.5c00839

[10] Ramakrishnan, R., Dral, P. O., Rupp, M. & von Lilienfeld, O. A. Quantum chemistry structures and properties of 134 kilo molecules. *Scientific Data* **1**, 140022 (2014). 25977779 10.1038/sdata.2014.22 PMC4322582

[11] Wiley Science Solutions. Wiley smartspectra ir database collection. Annual subscription spectral database (2024). Comprises approximately 250,000 predicted IR spectra validated against empirical libraries.

[12] Alberts, M., Laino, T. & Vaucher, A. Leveraging infrared spectroscopy for automated structure elucidation. *Communications Chemistry* **7**, 268 (2024). 39550488 10.1038/s42004-024-01341-w PMC11569215

[13] Krishnadas, A., Kansal, J., Charron, N. E. & Ragyanszki, A. SQuIRL: Spectra Quantum Chemistry Infrared and Resonance Library (2026).10.1038/s41597-026-07240-042000748

[14] Frisch, M. J. *et al*. *Gaussian 16 Revision C.01*. Gaussian Inc., Wallingford, CT (2016).

[15] Grimme, S. Accurate description of van der Waals complexes by density functional theory including empirical corrections. *Journal of Computational Chemistry* **25**(12), 1463–1473 (2004). 15224390 10.1002/jcc.20078

[16] Thom H. Dunning, J. Gaussian basis sets for use in correlated molecular calculations. i. the atoms boron through neon and hydrogen. *The Journal of Chemical Physics* **90**(2), 1007–1023 (1989).

[17] Laury, M. L., Carlson, M. J. & Wilson, A. K. Vibrational frequency scale factors for density functional theory and the polarization consistent basis sets. *Journal of Computational Chemistry* **33**(30), 2380–2387 (2012). 22815183 10.1002/jcc.23073

[18] Alecu, I. M., Zheng, J., Zhao, Y. & Truhlar, D. G. Computational thermochemistry: Scale factor databases and scale factors for vibrational frequencies obtained from electronic model chemistries. *Journal of Chemical Theory and Computation* **6**(9), 2872–2887 (2010). 26616087 10.1021/ct100326h

[19] Kesharwani, M. K., Brauer, B. & Martin, J. M. L. Frequency and zero-point vibrational energy scale factors for double-hybrid density functionals (and other selected methods): Can anharmonic force fields be avoided? *Journal of Physical Chemistry A* **119**(9), 1701–1714 (2015). 25296165 10.1021/jp508422u

[20] Mardirossian, N. & Head-Gordon, M. Thirty years of density functional theory in computational chemistry: An overview and extensive assessment of 200 density functionals. *Molecular Physics* **115**(19), 2315–2372 (2017).

[21] Purvis, G. D. & Bartlett, R. J. A full coupled-cluster singles and doubles model: The inclusion of disconnected triples. *The Journal of Chemical Physics* **76**(4), 1910–1918 (1982).

[22] NIST Mass Spectrometry Data Center & Wallace, W. E. Infrared spectra. In Linstrom, P. J. & Mallard, W. G. (eds.) *NIST Chemistry Webbook (NIST Standard Reference Database Number 69)* (National Institute of Standards and Technology, 2025). NIST SRD No. 69.

[23] Coblentz Society, Inc. Evaluated infrared reference spectra. In Linstrom, P. J. & Mallard, W. G. (eds.) *NIST Chemistry Webbook (NIST Standard Reference Database Number 69)* (National Institute of Standards and Technology, 2017). NIST SRD No. 69.

[24] Chu, P. M., Guenther, F. R., Rhoderick, G. C. & Lafferty, W. J. Quantitative infrared database. In Linstrom, P. J. & Mallard, W. G. (eds.) *NIST Chemistry Webbook (NIST Standard Reference Database Number 69)* (National Institute of Standards and Technology, 2025). NIST SRD No. 69.

[25] Dennington, R., Keith, T. A. & Millam, J. M. GaussView Version 6 (2019). Semichem Inc., Shawnee Mission KS.

[26] Shimanouchi, T. *Tables of Molecular Vibrational Frequencies*, vol. 1 of *National Standard Reference Data Series* (National Bureau of Standards, Gaithersburg, MD, 1972).

[27] Carbonnière, P., Lucca, T., Pouchan, C., Rega, N. & Barone, V. Vibrational computations beyond the harmonic approximation: Performances of the b3lyp density functional for semirigid molecules. *Journal of Computational Chemistry* **26**(4), 384–388 (2005). 15651031 10.1002/jcc.20170

[28] OpenBabel Project. SMARTS_InteLigand.txt. https://github.com/openbabel/openbabel/blob/master/data/SMARTS_InteLigand.txt (n.d.). Accessed: 2025-09-01.

[29] Landrum, G. Rdkit: Open-source cheminformatics. https://www.rdkit.org (2010).

